# Machine Learning Analysis of Cytotoxicity Determinants in Nanoparticle-Based Rheumatoid Arthritis Therapies

**DOI:** 10.1021/acs.molpharmaceut.5c00661

**Published:** 2025-10-23

**Authors:** Elif Yildirim, Irem Cakir, Nazar Ileri-Ercan

**Affiliations:** Chemical Engineering Department, 52984 Middle East Technical University, 06800 Ankara, Turkiye

**Keywords:** Nanoparticles, Machine Learning, Rheumatoid Arthritis

## Abstract

Nanoparticle-based therapies have gained attention in recent years as promising treatments for rheumatoid arthritis (RA), due to the potential offered for targeted delivery, controlled drug release, and improved biocompatibility. A deep understanding of the factors that drive cytotoxicity is crucial for safer and more effective nanomedicine formulations. To systematically analyze the determinants of cytotoxicity reported in the literature, we constructed a data set comprising 2,060 instances from 56 publications. Each instance was described by 23 features covering nanoparticle characteristics, cellular environment factors, and assay conditions potentially associated with cytotoxicity. Machine learning (ML) approaches were incorporated to gain deeper insight into key cytotoxicity drivers. We combined Boruta for feature selection, Random Forest (RF) for cytotoxicity prediction and feature importance evaluation, and Association Rule Mining (ARM) for rule-based, hidden pattern discovery. Boruta feature selection results identified the drug and nanoparticle concentration, core–shell material, and cell type as major determinants of cytotoxicity. The RF model demonstrated a strong predictive performance, further confirming the significance of these features. Moreover, ARM revealed high-confidence association rules linking specific conditions, such as high drug concentrations and poly­(aspartic acid)-based systems, to cytotoxic outcomes. This structured machine learning framework provides a foundation for optimizing nanoparticle formulations that balance therapeutic efficacy with cellular safety in RA therapy.

## Graphical abstract

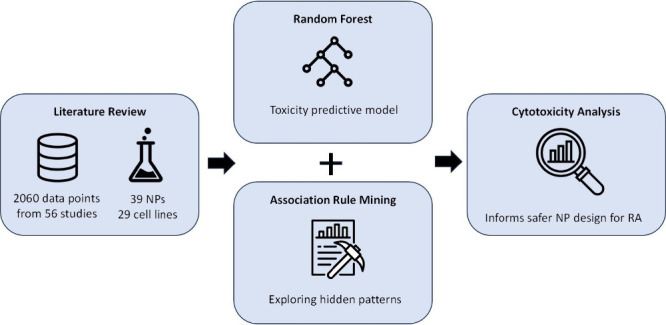

## Introduction

Rheumatoid arthritis (RA) is a chronic autoimmune disease characterized by joint inflammation, often resulting in persistent pain, swelling, and stiffness. Although RA primarily targets synovial linings of the joints, it can also affect other parts of the body such as the blood vessels, lungs, or heart. Both genetic and environmental factors influence the risk of developing RA.^1,2^ RA typically develops later in life and is more common in women than in men.^1,3^ By causing chronic inflammation and systemic complications, RA significantly reduces the quality of life for affected individuals.^4^ If left untreated, patients with RA may experience progressive joint damage, disability, and even life-threatening complications.^2^ According to the study of Global Burden of Disease (GBD), approximately 17.6 million people worldwide were affected by RA in 2020, and this number is projected to rise to 31.7 million by 2050.^5^

After years of investigation, the pathogenesis of RA remains not fully understood, and consequently, no definitive cure exists to date.^4^ The current treatment strategies for RA primarily include various painkillers and anti-inflammatory drugs aimed at relieving pain and inflammation, preserving joint structure, and improving patients’ quality of life.^4–7^ To date, there are no FDA-approved nanoparticle-based drug delivery systems for RA, with most developments remaining in the preclinical or clinical stages. The closest approved nanomedicine is certolizumab pegol (Cimzia), a PEGylated anti-TNFα antibody fragment.^8^

Nanoparticle (NP) therapy presents a promising alternative for RA treatment by offering several advantages over conventional approaches. The nanoscale size and modifiable surface properties enable effective penetration into targeted cells, allowing for targeted delivery to inflamed tissues, while minimizing accumulation in healthy cells. This reduces systemic toxicity and enhances biocompatibility.^9,10^ Nanoparticles also exhibit improved bioavailability and allow for controlled drug release.^9^ In recent years, numerous studies have proposed various nanoparticle-based treatments for RA. As the number of nanotreatment studies continues to grow, there is an increasing need to generalize the cytotoxicity behavior of previously synthesized nanoparticles in the field.^11,12^

Nanoparticles possess various physicochemical properties that significantly influence their biological interactions and potential cytotoxic effects.^13–17^ Key features that may affect cytotoxicity include nanoparticles’ material,^17^ size,^13–17^ shape,^13–17^ surface charge,^13,14,16,17^ concentration,^15,17^ exposure time,^15–17^ and synthesis method.^18,19^ Additionally, surface coatings,^15,17,19^ functional groups,^13,15–17^ and incorporated drugs^13,16^ can further enhance treatment efficiency. Moreover, the characteristics of the biological systems nanoparticles are tested,^14,17^ such as cell type, tissue origin, morphology, and source, as well as the conditions of the viability assays^17^ (e.g., assay type, assay indicator), also play a crucial role in determining cytotoxicity.

Given the diversity in material choice, surface modifications, dosages, and biological testing conditions across studies, making meaningful comparisons between results remains challenging. To gain a deeper understanding of how these features influence cytotoxicity, machine learning (ML) can serve as a valuable tool.^11,12^ In this study, we employed Boruta feature selection, Random Forest regression, and Association Rule Mining to systematically analyze the determinants of cytotoxicity. Boruta is a feature selection algorithm that assesses feature significance by comparing their importance against randomized shadow features.^20^ Random Forest (RF) is a supervised ML method that builds multiple decision trees and combines their outputs to make robust predictions and evaluate feature importance.^21^ On the other hand, Association Rule Mining (ARM) is an unsupervised learning technique that uncovers hidden patterns by identifying frequently co-occurring feature combinations.^22^ ARM and RF are commonly used in the cytotoxicity analysis of nanoparticles due to their ability to handle the complex, heterogeneous, and often nonlinear relationships present in toxicological data sets.^11,12^ These complementary ML approaches facilitate the generalization across diverse studies and provide a foundation for the efficient design of targeted nanomedicine strategies for RA. To this end, we constructed a data set comprising 2060 instances drawn from 56 articles. The data set includes 23 features related to nanoparticle characteristics, biological environment properties, and assay conditions. Corresponding cytotoxicity levels are also recorded for each instance. We applied ARM to uncover hidden patterns in this multidimensional data set and developed a predictive model using the Random Forest algorithm. The objective is to identify the most influential features contributing to nanoparticle toxicity and to provide insights that support the design of effective nanobased therapies for RA. This study offers a practical framework for comparing nanoparticle formulations across studies, to identify the most promising candidates for RA treatment.

## Materials and Methods

### Literature Survey and Data Set Formation

By the collection of information from 56 articles published between 2014 and 2024, a total of 2060 instances detailing in vitro nanoparticle cytotoxicity analyses were recorded. Google Scholar and Web of Science search engines were used to retrieve the articles, and inclusion criteria were based on the availability of quantitative cytotoxicity data (e.g., cell viability percentages) alongside detailed descriptions of nanoparticle properties, cell characteristics, and assay conditions. To ensure consistency across sources, units and terminologies for each feature were standardized. The features recorded in the data set are summarized in Figure 1. To isolate the independent effect of drugs on cytotoxicity, drug treatments without nanoparticle carriers were included as control groups. Nanoparticle-related features for these control groups were marked as “None” to ensure an accurate representation and prevent the introduction of incorrect data during subsequent imputation.

**Figure 1:**
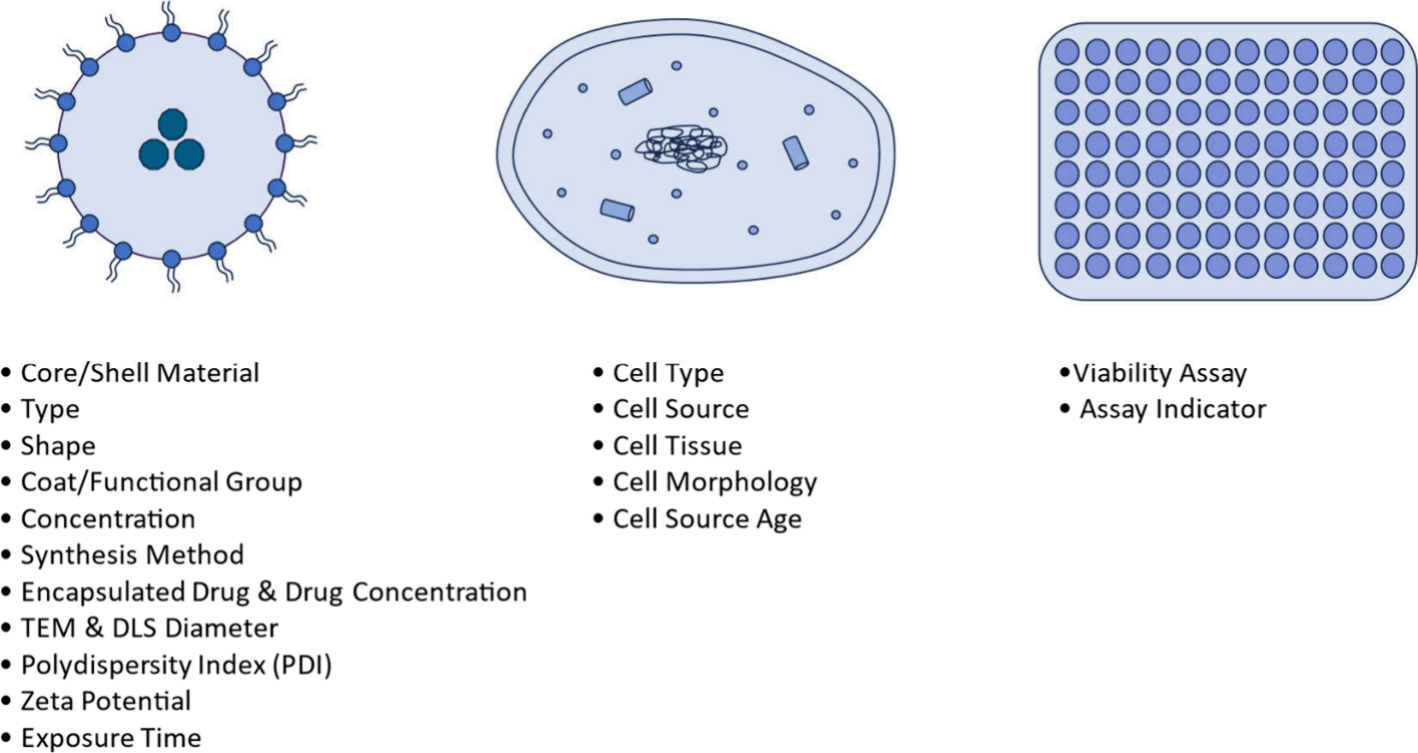
Features investigated in the data set related to nanoparticle characterization, cell properties, and assay conditions.

“Core and Shell Material” indicates the primary composition of the nanoparticle, while “Type” designation in the data set specifies whether the core nanoparticle material is derived from organic, inorganic, or hybrid sources. Surface modifications introduced to nanoparticles are recorded under the “Coat and Functional Group”. These are applied to modulate nanoparticle–cell interactions and, consequently, cytotoxic outcomes. Encapsulated drugs within the nanoparticle formulations are recorded in the data set, along with their respective concentrations. Both nanoparticle and drug concentrations are reported in micrograms per milliliter (μg/mL). Surface charge and zeta potential are captured separately: the former denotes the general charge polarity of the particle (positive or negative), while the latter provides a quantitative measure of the electrostatic potential at the nanoparticle surface in millivolts (mV). Particle sizes determined by Transmission Electron Microscopy (TEM) and Dynamic Light Scattering (DLS) are recorded in nanometers to capture both the shape and hydrodynamic size. Additional parameters such as the DLS medium and polydispersity index (PDI), which reflect size heterogeneity, are also included.

The cell types used in the experiments are identified by their scientific names along with details about their origin, such as source (human or animal), derived tissue, morphological characteristics, and biological age. Cytotoxicity assay conditions, including assay type and viability indicators, are specified. Cytotoxicity outcomes obtained from different assays (e.g., MTT, CCK-8) were incorporated into the data set only when expressed in relative terms (e.g., percentage viability). This standardized reporting scale provided a common base for direct comparability across studies, while reducing assay-specific variability and bias. Cell viability outcomes were extracted from graphs reported in articles using PlotDigitizer.^23^ Overall, the 23 features analyzed in this study were selected because they represent descriptors that are consistently and reliably reported across the literature.

### Model Development

All computational analyses for this study were performed using R.^24^ An imputation procedure was performed using the mice package^25^ to fill in the missing data based on generalized patterns in the data set before further analysis. Additionally, features providing bibliographic metadata such as data count, publication year, DOI, and article number were excluded from the data set. Feature selection was performed through the Boruta algorithm^26^ to identify the significance of features on cytotoxicity and potentially exclude those lacking significant contribution. Given its role as auxiliary information rather than as a core feature, the DLS medium was excluded from further analysis. Experiments performed under identical conditions but with varying drug or nanoparticle concentrations were grouped to capture their interdependence, thereby reducing data leakage and improving the relevance of the model to real-world scenarios.

A random forest regression model was constructed via the *randomForest* ^27^ package as a predictive model formulated to predict cell viability percentages. The data set was divided into training (80%) and test (20%) sets for model development and evaluation. Within the training set, model performance was further optimized using k-fold cross-validation, and final predictive accuracy was assessed on the test set by performance metric RMSE. Hyperparameter tuning was carried out through a grid search of the number of trees (ntree), the number of features at each split (mtry), and the number of folds (k) for cross validation, and the configuration yielding the lowest validation RMSE was selected as the optimal model. The final configuration involved 8-fold cross-validation with optimal hyperparameters identified as 350 trees and 8 features.

Following the implementation of the Random Forest model, Association Rule Mining (ARM) was employed utilizing the Apriori algorithm from the *arules* ^28^ package. ARM is implemented as a rule-based data mining method to identify obscure patterns linking specific features (antecedents) to cytotoxicity outcomes (consequents), categorized as either “cytotoxic” or “non-cytotoxic.” For ARM analysis, all numeric features were categorized based on their distributions and value ranges in the data set.

Following ISO 10993-5 guidelines, two separate ARM analyses were conducted based on different categorizations of cytotoxicity. In the first analysis, cell viability values greater than 70% were labeled as “non-cytotoxic,” while values below were classified as “cytotoxic.” In the second analysis, a more refined classification was applied to assess strong toxicity, where values were divided into four categories: strongly cytotoxic (≤40%), moderately cytotoxic (41–60%), weakly toxic (61–80%), and noncytotoxic (81–100%).

The performance of the association rules was assessed by the ARM evaluation metrics: support, confidence, and lift. To ensure the generation of reliable and meaningful rules, minimum support and confidence thresholds were set at 1% and 70%, respectively. Rules involving both single and double feature antecedents were explored to investigate the individual and combined effects on cytotoxicity. The concluding rules were ranked by highest lift to highlight the strongest associations.

## Results and Discussion

### Preliminary Analysis

A descriptive preliminary data set analysis was conducted before the detailed machine learning analysis. Among 2060 total data points, 1683 are related to the nanoparticles and 377 are related solely to the drug, which are the control groups. The frequency of nanoparticle features, such as the type, shape, PDI value, size, coat/functional group, synthesis method, concentration value, and surface charge are shown in Figure 2.

**Figure 2:**
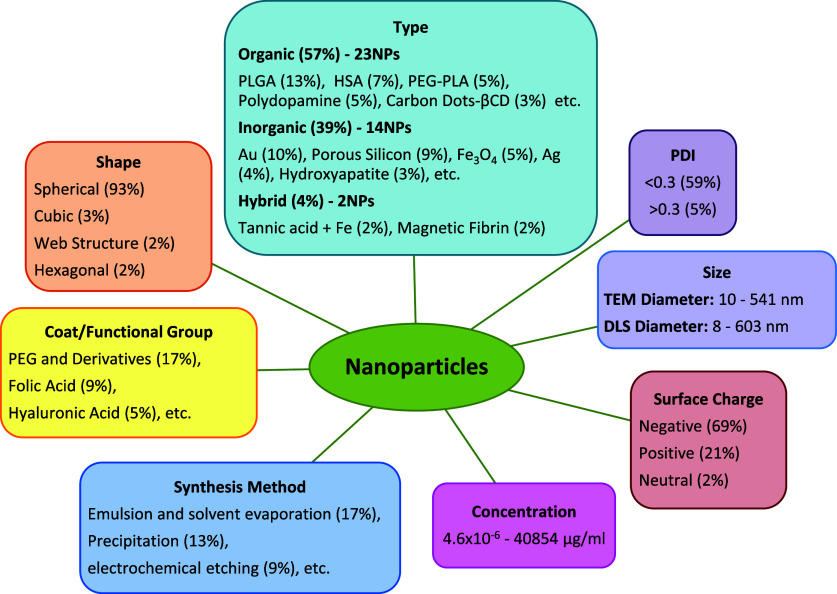
Frequencies of different nanoparticle features used in the data set.

There are 23 different organic-type nanoparticle materials, which cover 57% of total nanoparticles (946 data points) including PLGA (211 data points), Human Serum Albumin (125 data points), PEG3400-PLA2000 (90 data points), Polydopamine (84 data points), Carbon Dots-βCD (47 data points), etc.; 14 different inorganic-type nanoparticle materials, which cover 39% of the nanoparticles (661 data points) including Au (175 data points), porous silicon (149 data point), Fe_3_O_4_ (76 data points), Ag (59 data points), Hydroxyapatite (48 data points), etc.; and 2 different hybrid type nanoparticle materials which cover 4% of the nanoparticles (76 data points) including Tannic acid + Fe (36 data points), and magnetic fibrin (40 data points). Most of the used nanoparticles (92%) have spherical shapes (1556 data points), and the rest of the used nanoparticles (7%) have cubic shapes (61 data points), web structures (36 data points), and hexagonal shapes (30 data points). Additionally, among the nanoparticles for which a PDI value is provided, the majority have a PDI value lower than 0.3, with 1023 data points, while only the minority have a PDI value above 0.3, with 84 data points. The sizes range between 10 and 541 nm when measured by TEM, and between 8 and 603 nm when measured with DLS. The used concentration of nanoparticles ranges between 4.6 × 10^–6^ μg/mL and 40854 μg/mL. Moreover, among the nanoparticles of which the surface charges are measured, it is seen that 69% of the nanoparticles have a negative surface charge (1161 data points), 21% of the nanoparticles have a positive surface charge (354 data points) and 2% of the nanoparticles have neutral surface charge (30 data points). Examining the coat/functional group frequencies, it can be observed that PEG and its derivatives are the most preferred additives, with 286 data points, while folic acid (144 data points) and hyaluronic acid (77 data points) are the second and third most preferred additives, respectively. The synthesis method frequencies show that the most frequently used method is a combination of emulsion and solvent evaporation methods (283 data points) which is followed by the precipitation method (225 data points) and electrochemical etching method (149 data points).

In Figure 3, the frequency analysis of the drugs used is shown. In total, 22 different drugs were used in the experiments. The most frequently used drug for rheumatoid arthritis treatment is Methotrexate (MTX) and its derivatives with 523 data points. This is followed by Dexamethasone (DEX) with 112 data points and all-trans retinoic acid (ATRA) with 81 data points. There are a total of 1190 data points in which a drug is used, with 816 of them incorporated into the nanoparticles. The remaining data points are used as control groups without any nanoparticles.

**Figure 3:**
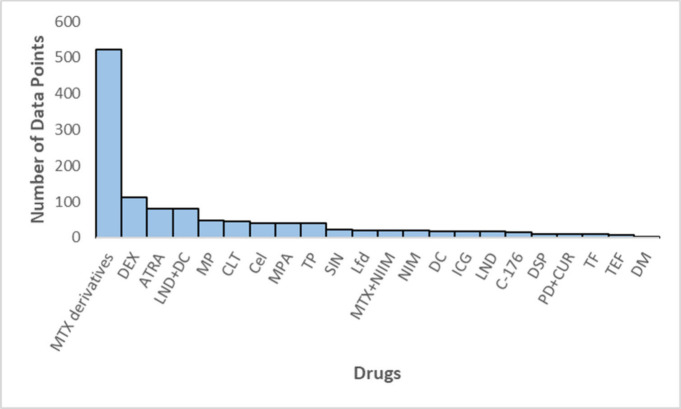
Data frequency analysis of the drugs.

The results for the analysis of cell type, cell source, cell activation, cell tissue, cell morphology, and source age are shown in Figure 4. A total of 17 different cell lines represent 68% of the data set (1409 data points), while 12 different primary cells account for 32% of the data set (651 data points). The most frequently used cell line is RAW 264.7 (823 data points), and the most commonly used primary cell type is HUVEC (126 data points). Approximately 22% of the total cells were activated by LPS (437 data points), TNF-α (5 data points), and IL-1β (3 data points). Additionally, animal-derived cells (1266 data points) are more commonly used than human-derived cells (794 data points). The tissue types from which the cells are sourced include blood (52%), synovial tissue (11%), and the umbilical vein (7%), among others. Regarding the morphology, the cells can be grouped as macrophage (46%), fibroblast (19%), epithelial (8%), and others. Moreover, the most used age group is adult (83%), followed by embryo (7%), neonatal (7%), and teen (3%).

**Figure 4:**
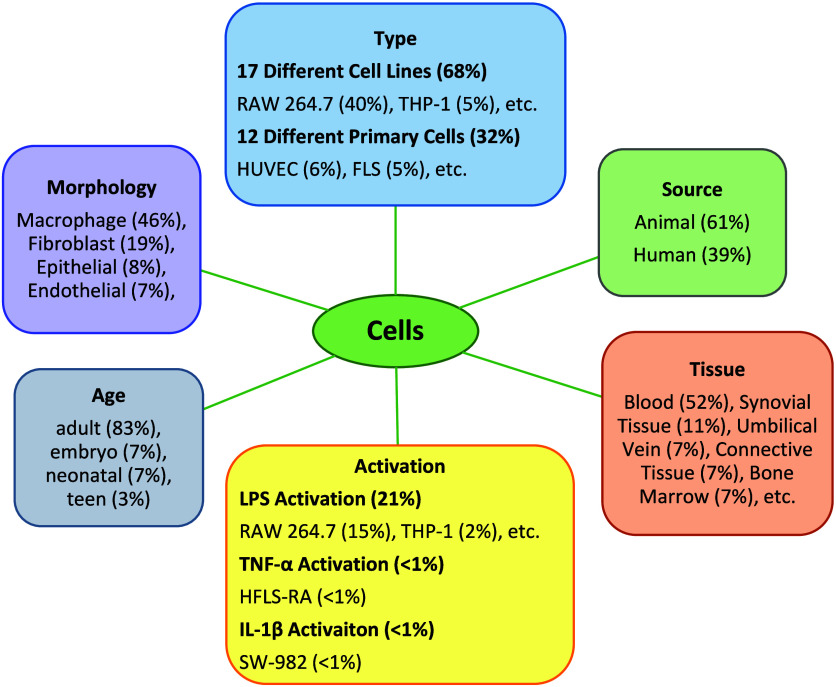
Frequencies of different cell features used in the data set.

Additional preliminary analysis can be conducted on the exposure time and the cell viability test used in the experiments included in the data set. The results of these analyses show that there are 9 different cell viability tests in total, with the majority of the data points using MTT (55%, 1134 data points), followed by CCK-8 (30%, 613 data points). Regarding the drug/nanoparticle exposure time, the most commonly preferred durations are 24 (59%), 48 (29%), and 72 h (7%). Additionally, 3% of the data used exposure time shorter than 24 h and 1% used exposure time longer than 72 h.

### Cytotoxicity Prediction with Random Forest

Boruta analysis was performed initially to identify the key features that should be retained for subsequent analysis. The results, shown in Figure 5, provided insights into the importance of features on cytotoxicity. No features were identified as insignificant determinants of cytotoxicity; therefore, all 23 features were retained for subsequent analysis.

**Figure 5:**
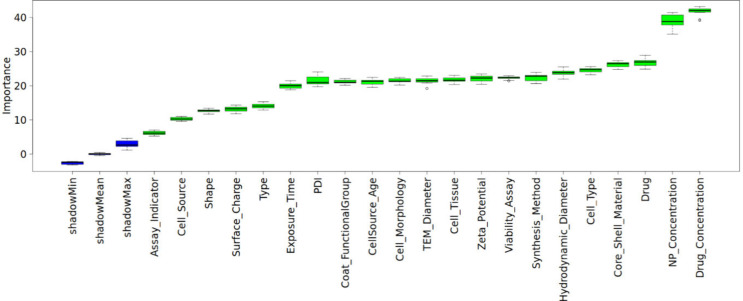
Boruta feature importance analysis.

Following feature selection, a Random Forest regression model was developed to predict cell viability outcomes. Figure 6 illustrates the model’s performance on training, validation, and test sets through their respective RMSE values, along with the intrinsic feature importance analysis generated by the Random Forest algorithm. High prediction accuracy was achieved on the training set (RMSE: 6.6), demonstrating the model’s ability to learn complex patterns in the data. RMSE values for the validation (18.1) and test (16.0) sets showed a marginal but expected increase, reflecting generalization to unseen data, while remaining within an acceptable range.

**Figure 6:**
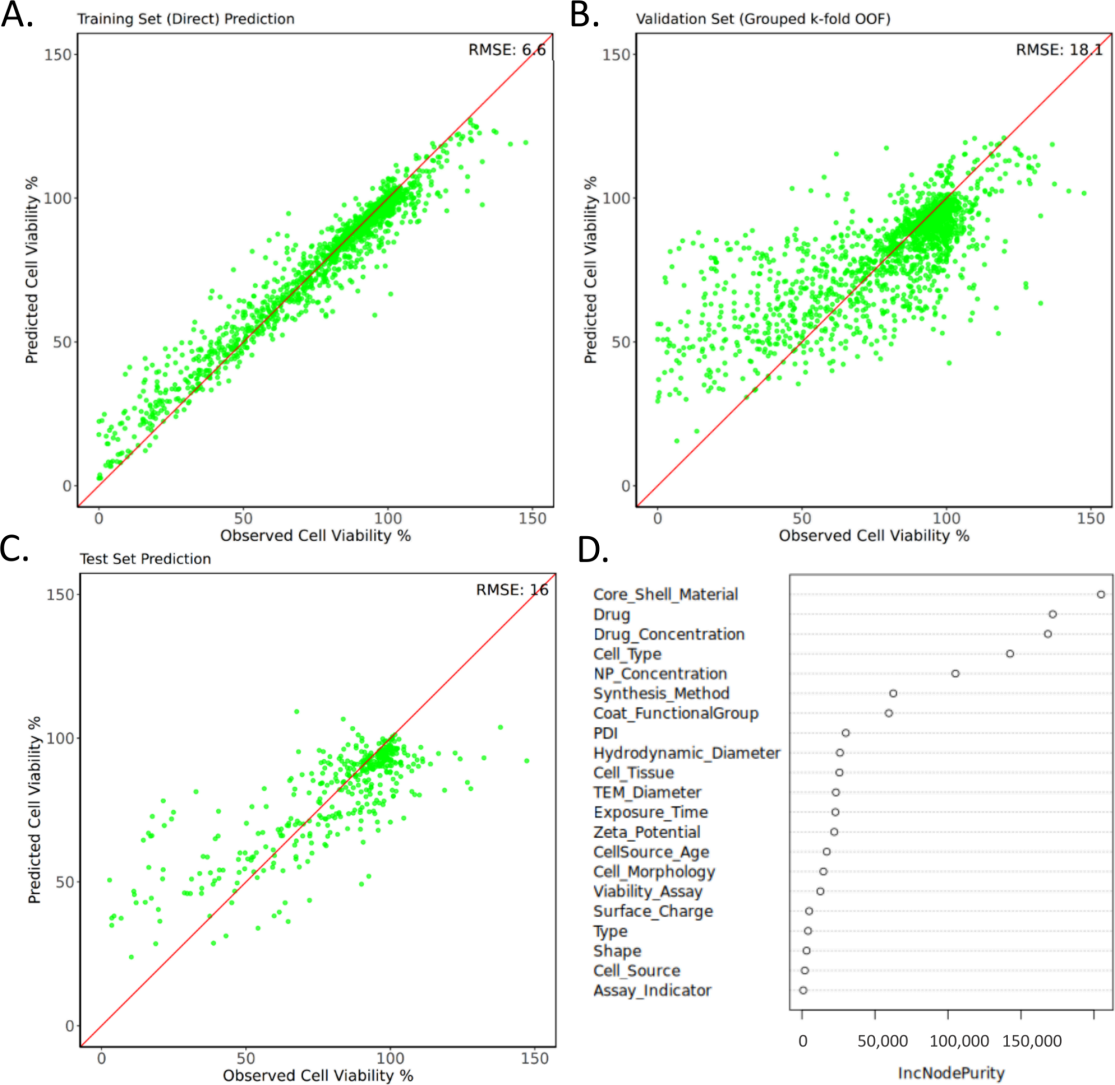
RF model prediction in (a) training, (b) validation, and (c) test sets along with (d) feature importance results.

A strong agreement between predicted and observed viability was observed in all sets, with most data points closely aligned along the *y = x* line, particularly in the training set. However, an overestimation of predicted cell viability values below 50% was the most notable in the test set. This may be attributed to class imbalance in the data set, where high viability samples are more prevalent and thus exert greater influence on the model. Additionally, experimental variabilities and the absence of features specifically related to low viability outcomes may also contribute to this discrepancy.

To complement the Boruta feature selection analysis, Random Forest feature importance rankings were also examined (Figure 6d) to gain additional insights into the contribution of individual descriptors to cytotoxicity. According to the RF model, the nanoparticle material emerged as the primary determinant of cytotoxicity, followed by the drug type and concentration. Cell types, nanoparticle concentrations, and synthesis methods also showed high importance, consistent with Boruta analysis. Overall, the feature importance results from RF largely aligned with those from Boruta, with minor differences attributed to methodological differences in how each algorithm evaluates the feature importance.

### Uncovering Cytotoxicity Patterns by Association Rule Mining

A deeper rule-based analysis was carried out via ARM to identify how specific features contribute to cytotoxic outcomes. The results for noncytotoxic outcomes are available in the . The resulting single-factor association rules linked to cytotoxic outcomes are summarized in Table 1. Support, confidence, and lift are performance metrics that characterize association rules. Support reflects the frequency of a rule appearing in the data set, confidence represents the probability of the rule being true, and lift measures the strength of the association compared to random chance. While a lift value of 1 indicates no association, greater values indicate stronger associations. The rules were ranked in decreasing order of lift and evaluated by using the performance metrics to assess the strength and reliability of the associations.

**Table 1:** Single-Factor Rules Associated with Cytotoxic Outcomes^a^

Support	Confidence	Lift	Count	Antecedent	Consequent
0.0131	0.9643	3.6	27*	{Core_Shell_Material = poly(aspartic acid)}	{Cell_Viability = cytotoxic}
0.0146	0.8333	3.1	30*	{Core_Shell_Material = HA}	{Cell_Viability = cytotoxic}
0.0311	0.7901	2.9	64*	{Drug = LND+DC}	{Cell_Viability = cytotoxic}
0.0146	0.7500	2.8	30*	{Drug = MPA}	{Cell_Viability = cytotoxic}
0.0388	0.7080	2.6	80*	{Cell_Types = FLS}	{Cell_Viability = cytotoxic}

a Confidence ≥ 70%; asterisks indicate data sourced from a single study.

While each single-factor association stems from a single study, similar patterns have been described in the literature to support their plausibility and relevance. Still, these findings should be interpreted with caution, as further validation is required to confirm their reliability. The strongest single-factor association rule resulting in cytotoxic outcomes was observed for poly­(aspartic acid), utilized as the core–shell material (confidence = 96.4%, lift = 3.6). Interestingly, HLA-DRB1 alleles encoding aspartic acid at position 70 have been associated with a reduced risk of developing rheumatoid arthritis,^29–31^ suggesting that aspartic acid may possess immunomodulatory properties that could influence cellular responses to poly­(aspartic acid). The observed association between poly­(aspartic acid)-based nanomaterials and cytotoxic outcomes may be related to the molecular presentation of aspartic acid residues, which could modulate immune-related cellular responses. Furthermore, isoaspartate formation has been shown to trigger autoimmune responses to self-proteins,^32^ implying that structural variations or mimetics of aspartic acid residues could alter immune recognition. This may help contextualize the cytotoxic association observed with poly­(aspartic acid)-based nanomaterials, particularly in inflammatory or immunologically active environments.

Hyaluronic acid, when used as the primary material in a nanomedicine, is associated with increased toxicity (confidence = 83.3%, lift = 3.1). Although hyaluronic acid (HA) is widely reported as nontoxic and biocompatible,^33–36^ there are documented instances where HA conjugation has led to enhanced cytotoxicity, potentially due to increased cellular uptake and elevated intracellular drug concentrations.^37^ Additionally, HA fragments with low molecular weights have been reported to exhibit pro-inflammatory effects.^38^ When used as a bulk in the core/shell of a nanomedicine, high concentrations of HA can degrade, especially if enzymes such as hyaluronidase are present, which is common in RA environments. Hence, this may indicate multimodal sensitivity, where the cytotoxic effects of HA are influenced by additional important factors that influence certain mechanisms in nanomedicine.

The combination of Lonidamine (LND) and Diclofenac (DC) drugs utilized in a nanomedicine to treat RA has shown strong associations with cytotoxicity (confidence = 79.0%, lift = 2.9). LND has been reported in the literature to reduce inflammatory injury in arthritis models,^39^ induce apoptosis in rheumatoid arthritis synovial fibroblasts (RASFs),^40^ and trigger mitochondrial-mediated cytotoxicity in cancer cells.^41^ While Lonidamine alone has been reported to exhibit minimal cytotoxicity at low concentrations, increased toxicity is observed at higher doses, particularly when concentrations exceed 100 μM.^42,43^ LND has also been shown to enhance the cytotoxic effects of various antineoplastic drugs.^42,44^ In contrast, Diclofenac (DC), a commonly used nonsteroidal anti-inflammatory drug (NSAID), is well-known for its dose-dependent hepatotoxicity.^45–47^ The strong association between the LND+DC drug combination and cytotoxic outcomes may therefore reflect synergistic or additive toxicity, particularly at high concentrations.

Additionally, methylprednisolone acetate (MPA) utilized as a drug also showed high cytotoxicity (confidence = 75.0%, lift = 2.8). The original study from which this rule was derived reported reduced cytotoxicity for MPA-loaded nanoparticles compared to the pure drug,^48^ and previous literature supports MPA’s therapeutic role in alleviating inflammation symptoms of RA.^49,50^ This discrepancy suggests that formulation, dosage, or experimental characteristics may significantly influence cytotoxicity. Lastly, the utilization of cell type FLS in cytotoxicity assays also commonly resulted in cytotoxic outcomes (confidence = 70.8%, lift = 2.6). FLS cells are key effector cells in RA^51^ and may exhibit increased sensitivity to cytotoxic agents, which could potentially explain their frequent association with cytotoxic outcomes in inflammatory contexts.

Double-factor association rules resulting in cytotoxic outcomes are summarized and ranked by lift in Table 2. Poly­(aspartic acid) consistently appeared as a strong determinant in cytotoxicity across multiple high-confidence rules in combination with features such as large hydrodynamic diameter (>150 nm), LPS-activated RAW264.7 cells, encapsulated MTX, weakly negative zeta potentials between −20 and 0 mV, or exposure times between 24 and 48 h. Similarly, combinations involving high drug concentrations above 100 μg/mL were observed with LND+DC drug combination and FLS cell lines. This could further highlight the cytotoxic effects of the synergistic LND+DC drug system at high concentrations. LND+DC drug systems with high nanoparticle concentrations, along with TEM diameters between 50 and 150 nm, also show strong associations with cytotoxicity. The assembly synthesis method was repeatedly linked to cytotoxic outcomes, particularly when combined with high nanoparticle concentrations, LPS-activated RAW 264.7 cells, hydrodynamic diameters above 150 nm, MTX drug, or weakly negative zeta potentials. The nanoparticle materials synthesized by the assembly method in the data set include hyaluronic acid, poly­(aspartic acid), poly­(β-benzyl-l-aspartate), dextran and all-trans retinal, human serum albumin, saponin, and bilirubin. Most of the nanoparticles were based on polymeric materials, particularly natural polysaccharides such as hyaluronic acid and dextran, and synthetic polypeptides such as poly­(aspartic acid and poly­(β-benzyl-l-aspartate). In addition, high drug concentrations combined with no core/shell material, indicating control groups where cells are treated solely by drugs, resulted in cytotoxic outcomes. High nanoparticle concentrations targeting synovial tissues are also associated with cytotoxicity. Triptolide-loaded nanoparticles exhibiting TEM diameters between 50 and 150 nm are also associated with cytotoxic outcomes. This finding is consistent with existing literature, where triptolide is widely reported for its toxicity and narrow therapeutic window.^52–55^ The resulting strong association rule may reflect both the inherent toxicity and the size dependency of triptolide, which modulates the cellular uptake efficiency and therefore may amplify cytotoxic effects. Cytotoxic outcomes are observed with LPS-activated RAW264.7 murine macrophage cell lines combined with several factors such as medium drug concentration, hydrodynamic diameters between 50 and 150 nm, TEM diameters above 150 nm, and positively charged particles. Positively charged nanoparticles are generally reported in the literature as showing cytotoxic behavior than negatively charged nanoparticles.^13,14,16^ Lastly, the commonly used RA drug MTX showed cytotoxicity with exposure times between 24 and 48 h.

**Table 2:** Double-Factor Rules Associated with Cytotoxic Outcomes^a^

Support	Confidence	Lift	Count	Antecedent	Consequent
0.0102	1.0000	3.7	21*	{Core_Shell_Material = poly(aspartic acid), Hydrodynamic Diameter = above 150}	{Cell_Viability = cytotoxic}
0.0155	1.0000	3.7	32*	{Drug = LND+DC, Drug_Concentration = high}	{Cell_Viability = cytotoxic}
0.0175	1.0000	3.7	36*	{Drug Concentration = high, Cel_Type = FLS}	{Cell_Viability = cytotoxic}
0.0131	0.9643	3.6	27*	{Core_Shell_Material = poly(aspartic acid), Cell_Type = LPS activated RAW 264.7}	{Cell_Viability = cytotoxic}
0.0131	0.9643	3.6	27*	{Core_Shell_Material = poly(aspartic acid), Drug = MTX}	{Cell_Viability = cytotoxic}
0.0131	0.9643	3.6	27*	{Core_Shell_Material = poly(aspartic acid), Exposure_Time = 24–48}	{Cell_Viability = cytotoxic}
0.0131	0.9643	3.6	27*	{Core_Shell_Material = poly(aspartic acid), Zeta_Potential = weakly negative}	{Cell_Viability = cytotoxic}
0.0121	0.9615	3.6	25*	{Drug = LND+DC, TEM_Diameter = 50–150}	{Cell_Viability = cytotoxic}
0.0165	0.9714	3.6	34*	{Synthesis_Method = assembly method, Cell_Type = LPS activated RAW 264.7}	{Cell_Viability = cytotoxic}
0.0189	0.9750	3.6	39	{Drug = MTX, Synthesis_Method = assembly method}	{Cell_Viability = cytotoxic}
0.0141	0.9667	3.6	29	{Synthesis_Method = assembly method, NP_Concentration = high}	{Cell_Viability = cytotoxic}
0.0257	0.9298	3.5	53	{Core_Shell_Material = None, Drug_Concentration = high}	{Cell_Viability = cytotoxic}
0.0170	0.8750	3.3	35*	{Drug = LND+DC, NP_Concentration = high}	{Cell_Viability = cytotoxic}
0.0228	0.8545	3.2	47*	{Cell_Type = FLS,NP_Concentration = high}	{Cell_Viability = cytotoxic}
0.0194	0.8696	3.2	40	{Synthesis_Method = assembly method, Hydrodynamic_Diameter = above 150}	{Cell_Viability = cytotoxic}
0.0311	0.7901	2.9	64*	{Drug = LND+DC, Cell_Type = FLS}	{Cell_Viability = cytotoxic}
0.0228	0.7705	2.9	47*	{Synthesis_Method = assembly method, Zeta_Potential = weakly negative}	{Cell_Viability = cytotoxic}
0.0335	0.7841	2.9	69	{Cell_Tissue = Synovial Tissue, NP_Concentration = high}	{Cell_Viability = cytotoxic}
0.0252	0.7647	2.8	52*	{Drug_Concentration = high, Hydrodynamic_Diameter = 50–150}	{Cell_Viability = cytotoxic}
0.0102	0.7500	2.8	21	{Drug = TP, TEM_Diameter = 50–150}	{Cell_Viability = cytotoxic}
0.0136	0.7568	2.8	28	{Synthesis_Method = assembly method, TEM_Diameter = 50–150}	{Cell_Viability = cytotoxic}
0.0510	0.7664	2.8	105	{Drug = MTX, Cell_Type = LPS activated RAW 264.7}	{Cell_Viability = cytotoxic}
0.0155	0.7273	2.7	32*	{Zeta_Potential = weakly negative, Cell_Types = FLS}	{Cell_Viability = cytotoxic}
0.0398	0.7257	2.7	82*	{Drug_Concentration = medium, Cell_Type = LPS activated RAW 264.7}	{Cell_Viability = cytotoxic}
0.0398	0.7130	2.7	82*	{Hydrodynamic_Diameter = 50–150, Cell_Type = LPS activated RAW 264.7}	{Cell_Viability = cytotoxic}
0.0146	0.7143	2.7	30	{Surface_Charge = Positive, Cell_Type = LPS activated RAW 264.7}	{Cell_Viability = cytotoxic}
0.0218	0.7143	2.7	45	{TEM_Diameter = above 150, Cell_Type = LPS activated RAW 264.7}	{Cell_Viability = cytotoxic}
0.0398	0.7257	2.7	82	{Drug_Concentration = medium, Cell_Type = LPS activated RAW 264.7}	{Cell_Viability = cytotoxic}
0.0490	0.7063	2.6	101	{Drug = MTX, Exposure_Tine = 24–48}	{Cell_Viability = cytotoxic}

a Confidence ≥ 70%; asterisks indicate data sourced from a single study.

The double factor association rules from the second analysis, investigating for high cytotoxicity (cell viability <40%), are presented in Table 3, ranked by their lift values. The rules tabulated in Table 3 are all derived from multiple sources. Strong cytotoxic outcomes are dominated by high drug concentrations above 100 μg/mL and are influenced by data entries combined with the corresponding concentration range. The lift values for strong cytotoxic rules are exceptionally high (between 6 and 9), highlighting how strongly high-dose exposure of drugs predicts a severe loss of viability. High drug concentrations are combined with the combined LND and DC drug system, FLS cells, cells derived from synovial tissues, and fibroblast cells along with MTT viability assays. The high drug concentrations in control groups without core/shell materials confirm that the drug concentration alone can be sufficient to induce significant cell death.

**Table 3:** Double-Factor Rules Resulting in Strong Cytotoxicity^a^

Support	Confidence	Lift	Count	Antecedent	Consequent
0.0155	1.0000	9	32	{Drug = LND+DC, Drug_Concentration = high}	{Cell_Viability = strongly cytotoxic}
0.0170	0.9722	8.8	35	{Drug_Concentration = high, Cell_Type = FLS}	{Cell_Viability = strongly cytotoxic}
0.0170	0.9211	8.3	35	{Drug_Concentration = high, Cell_Tissue = Synovial Tissue}	{Cell_Viability = strongly cytotoxic}
0.0194	0.8333	7.5	40	{Drug_Concentration = high, Cell_Morphology = Fibroblast}	{Cell_Viability = strongly cytotoxic}
0.0243	0.7692	7	50	{Drug_Concentration = high, Viability_Assay = MTT}	{Cell_Viability = strongly cytotoxic}
0.0199	0.7193	6.5	41	{Core_Shell_Material = None, Drug_Concentration = high}	{Cell_Viability = strongly cytotoxic}
0.0199	0.7193	6.5	41	{Type = None, Drug_Concentration = high}	{Cell_Viability = strongly cytotoxic}
0.0199	0.7193	6.5	41	{Shape = None, Drug_Concentration = high}	{Cell_Viability = strongly cytotoxic}
0.0199	0.7193	6.5	41	{Drug_Concentration = high, Synthesis_Method = None}	{Cell_Viability = strongly cytotoxic}
0.0199	0.7193	6.5	41	{Drug_Concentration = high, Surface_Charge = None}	{Cell_Viability = strongly cytotoxic}
0.0136	0.7000	6.3	28	{Drug = LND+DC, NP_Concentration = high}	{Cell_Viability = strongly cytotoxic}

a Confidence ≥ 70%.

## Conclusion

By integrating Boruta feature selection, Random Forest cytotoxicity prediction, and ARM hidden pattern discovery, this study presents a multistep machine learning framework to support the design of more effective nanoparticles in RA therapy. Boruta analysis revealed that all features in the data set contributed significantly to cytotoxicity, and thus, no features were excluded from the data set. The Random Forest model demonstrated strong predictive performance, which confirms its ability to capture patterns related to cytotoxicity in the data set. In addition, the feature importance analyses conducted by Boruta and Random Forest were largely consistent, as both highlighted the primary features affecting cytotoxicity as drug and core/shell materials, drug and NP concentrations, and cell types. ARM analysis uncovered meaningful association rules with high confidence and lift values by analyzing both single- and double factor associations to capture the individual and combined effects of features. High nanoparticle concentrations along with specific material and drug choices were associated with cytotoxic outcomes. Control groups involving only drug treatments and high drug concentrations frequently resulted in association rules with toxic outcomes with high lift. Moreover, strong cytotoxicity-focused analysis confirmed the high drug concentration as a dominant factor. To reduce the risk of multicollinearity influencing feature importance, Boruta feature selection was complemented by the internal feature importance rankings of the Random Forest. In addition, both single and double factor association rules were generated during ARM, enabling us to examine not only individual effects but also potential synergistic interactions between features. In conclusion, these findings identify key features influencing cytotoxicity and suggest critical design considerations for developing more effective and biocompatible nanoparticle formulations for the treatment of RA.

## Supplementary Material





## Data Availability

The data set is available in the .

## Notes

Single-factor and double-factor rules associated with noncytotoxic outcomes (PDF)
Data set (XLSX)

## References

[1] Finckh A., Gilbert B., Hodkinson B. (2022). Global epidemiology
of rheumatoid arthritis. Nat. Rev. Rheumatol.

[2] Gao Y., Zhang Y., Liu X. (2024). Rheumatoid arthritis: pathogenesis
and therapeutic advances. MedComm (2020).

[3] Uke P., Maharaj A., Adebajo A. (2025). A review on the epidemiology of rheumatoid
arthritis: An update and trends from current literature. Best Pract Res. Clin Rheumatol..

[4] An X., Yang J., Cui X., Zhao J., Jiang C., Tang M., Dong Y., Lin L., Li H., Wang F. (2024). Advances in local drug delivery technologies for improved rheumatoid
arthritis therapy. Adv. Drug Delivery Rev..

[5] GBD
2021 Rheumatoid Arthritis Collaborators (2023). Global, regional, and national burden of rheumatoid
arthritis, 1990–2020, and projections to 2050: a systematic
analysis of the Global Burden of Disease Study 2021. Lancet Rheumatol..

[6] Smolen J. S, Landewe R. B M, Bijlsma J. W J, Burmester G. R, Dougados M., Kerschbaumer A., McInnes I. B, Sepriano A., van Vollenhoven R. F, de Wit M., Aletaha D., Aringer M., Askling J., Balsa A., Boers M., den Broeder A. A, Buch M. H, Buttgereit F., Caporali R., Cardiel M. H., De Cock D., Codreanu C., Cutolo M., Edwards C. J., van Eijk-Hustings Y., Emery P., Finckh A., Gossec L., Gottenberg J.-E., Hetland M. L., Huizinga T. W J, Koloumas M., Li Z., Mariette X., Muller-Ladner U., Mysler E. F, da Silva J. A P, Poor G., Pope J. E, Rubbert-Roth A., Ruyssen-Witrand A., Saag K. G, Strangfeld A., Takeuchi T., Voshaar M., Westhovens R., van der Heijde D. (2020). EULAR recommendations for the management of rheumatoid
arthritis with synthetic and biological disease-modifying antirheumatic
drugs: 2019 update. Ann. Rheum Dis.

[7] McGonagle D., Watad A., Savic S. (2018). Mechanistic
immunological based classification
of rheumatoid arthritis. Autoimmun Rev..

[8] Patra J. K., Das G., Fraceto L. F. (2018). Nano based drug delivery systems: recent
developments and future prospects. J. Nanobiotechnol.

[9] Angela S., Fadhilah G., Hsiao W. W.-W., Lin H.-Y., Ko J., Lu S. C.-W., Lee C.-C., Chang Y.-S., Lin C.-Y., Chang H.-C., Chiang W.-H. (2024). Nanomaterials
in the treatment and
diagnosis of rheumatoid arthritis: Advanced approaches. SLAS Technology.

[10] An X., Yang J., Cui X., Zhao J., Jiang C., Tang M., Dong Y., Lin L., Li H., Wang F. (2024). Advances in local drug delivery technologies
for improved rheumatoid
arthritis therapy. Adv. Drug Delivery Rev..

[11] Genc D. E., Ozbek O., Oral B., Yıldırım R., İleri Ercan N. (2024). Phytochemicals
in Pancreatic Cancer Treatment: A Machine
Learning Study. ACS Omega.

[12] Gul G., Yildirim R., İLERİ
ERCAN N. (2021). Cytotoxicity analysis
of nanoparticles by association rule mining. Environmental Science. Nano.

[13] Shin S. W., Song I. H., Um S. H. (2015). Role of physicochemical properties
in nanoparticle toxicity. Nanomaterials.

[14] Rivera-Gil P., Jimenez De Aberasturi D., Wulf V., Pelaz B., Del Pino P., Zhao Y., De La Fuente J. M., Ruiz De Larramendi I., Rojo T., Liang X.-J., Parak W. J. (2013). The challenge
to relate the physicochemical properties of colloidal nanoparticles
to their cytotoxicity. Acc. Chem. Res..

[15] Li X., Liu W., Sun L., Aifantis K. E., Yu B., Fan Y., Feng Q., Cui F., Watari F. (2015). Effects of physicochemical
properties of nanomaterials on their toxicity. J. Biomed. Mater. Res., Part A.

[16] Huang Y. W., Cambre M., Lee H. J. (2017). The toxicity
of nanoparticles depends
on multiple molecular and physicochemical mechanisms. International journal of molecular sciences.

[17] Labouta H. I., Asgarian N., Rinker K., Cramb D. T. (2019). Meta-analysis of
nanoparticle cytotoxicity via data-mining the literature. ACS Nano.

[18] de
Lima R., Seabra A. B., Durán N. (2012). Silver nanoparticles: a brief review
of cytotoxicity and genotoxicity of chemically and biogenically synthesized
nanoparticles. Journal of Applied Toxicology.

[19] Odaudu, O. R. ; Akinsiku, A. A. (2022, September). Toxicity and cytotoxicity effects of selected nanoparticles: a review. In IOP Conference Series: Earth and Environmental Science (Vol. 1054, No. 1, p. 012007). IOP Publishing.

[20] Kursa M. B., Rudnicki W. R. (2010). Feature selection
with the Boruta package. J. Stat. Soft..

[21] Rigatti S. J. (2017). Random
forest. Journal of Insurance Medicine.

[22] Kumbhare T. A., Chobe S. V. (2014). An overview of association rule mining algorithms. International Journal of Computer Science and Information
Technologies.

[23] PlotDigitizer , 3.1.6, 2025, https://plotdigitizer.com (accessed 2025-02-18).

[24] R Core Team (2023). “R: A Language and Environment for Statistical Computing”. R Foundation for Statistical Computing: Vienna, Austria. https://www.R-project.org/ (accessed 2025-03-10).

[25] van
Buuren S., Groothuis-Oudshoorn K. (2011). mice: Multivariate Imputation by
Chained Equations in R. Journal of Statistical
Software.

[26] Kursa M. B., Rudnicki W. R. (2010). Feature Selection with the Boruta Package. Journal of Statistical Software.

[27] Breiman L. (2001). Random Forests. Mach. Learn..

[28] Hahsler M., Grü n B., Hornik K. (2005). Arules - A Computational Environment
for Mining Association Rules and Frequent Item Sets. J. Stat. Software.

[29] Mattey D. L., Dawes P. T., Gonzalez-Gay M. A., Garcia-Porrua C. A. R. L. O. S., Thomson W. E. N. D. Y., Hajeer A. H., Ollier W. E. (2001). HLA-DRB1
alleles encoding an aspartic acid at position 70 protect against development
of rheumatoid arthritis. Journal of rheumatology.

[30] Ruiz-Morales J. A., Vargas-Alarcón G., Flores-Villanueva P. O., Villarreal-Garza C., Hernández-Pacheco G., Yamamoto-Furusho J. K., Rodríguez-Pérez J. M., Pérez-Hernández N., Rull M., Cardiel M. H., Granados J. (2004). HLA-DRB1 alleles encoding
the ″shared epitope″ are associated with susceptibility
to developing rheumatoid arthritis whereas HLA-DRB1 alleles encoding
an aspartic acid at position 70 of the beta-chain are protective in
Mexican Mestizos. Hum. Immunol..

[31] de
Vries N., Tijssen H., van Riel P. L. C. M., van de Putte L. B. A. (2002). Reshaping the shared epitope hypothesis: HLA-associated
risk for rheumatoid arthritis is encoded by amino acid substitutions
at positions 67–74 of the HLA–DRB1 molecule. Arthritis & Rheumatism.

[32] Mamula M. J., Gee R. J., Elliott J. I., Sette A., Southwood S., Jones P. J., Blier P. R. (1999). Isoaspartyl
post-translational modification
triggers autoimmune responses to self-proteins. J. Biol. Chem..

[33] Kim K. S., Park S. J., Yang J.-A., Jeon J.-H., Bhang S. H., Kim B.-S., Hahn S. K. (2011). Injectable hyaluronic
acid–tyramine
hydrogels for the treatment of rheumatoid arthritis. Acta Biomaterialia.

[34] Mirchandani Y., Patravale V. B., Brijesh S. (2022). Hyaluronic Acid-Coated Solid Lipid
Nanoparticles Enhance Antirheumatic Activity and Reduce Toxicity of
Methotrexate. Nanomedicine.

[35] Marinho A., Nunes C., Reis S. (2021). Hyaluronic
Acid: A Key Ingredient
in the Therapy of Inflammation. Biomolecules..

[36] Zhou M., Hou J., Zhong Z., Hao N., Lin Y., Li C. (2018). Targeted delivery
of hyaluronic acid-coated solid lipid nanoparticles for rheumatoid
arthritis therapy. Drug Delivery.

[37] Gouveia V. M., Lopes-de-Araújo J., Costa Lima S. A., Nunes C., Reis S. (2018). Hyaluronic acid-conjugated pH-sensitive
liposomes for targeted delivery of prednisolone on rheumatoid arthritis
therapy. Nanomedicine (Lond)..

[38] You N., Chu S., Cai B., Gao Y., Hui M., Zhu J., Wang M. (2021). Bioactive hyaluronic
acid fragments inhibit lipopolysaccharide-induced
inflammatory responses via the Toll-like receptor 4 signaling pathway. Front Med..

[39] Chen C., Zhou Y., Ning X., Li S., Xue D., Wei C., Yin W. (2022). Directly targeting ASC by lonidamine alleviates inflammasome-driven
diseases. Journal of neuroinflammation.

[40] Song G., Lu Q., Fan H., Zhang X., Ge L., Tian R., Wang S., Feng T., Pan J., Feng J., Xiao Y., Yi X., Ren N., Wang L. (2019). Inhibition
of hexokinases holds potential as treatment strategy for rheumatoid
arthritis. Arthritis Res. Ther..

[41] Ravagnan L., Marzo I., Costantini P., Susin S. A., Zamzami N., Petit P. X., Hirsch F., Goulbern M., Poupon M. F., Miccoli L., Xie Z., Reed J. C., Kroemer G. (1999). Lonidamine
triggers apoptosis via a direct, Bcl-2-inhibited effect on the mitochondrial
permeability transition pore. Oncogene..

[42] Hinrichs, C. (2015). Exploring the anti-leukemic effect of the combination treatment with Valproic acid, Lonidamine and Mycophenolate mofetil in acute myeloid leukemia (Master’s thesis, The University of Bergen).

[43] Forster R., Campana A., D’Onofrio E., Henderson L., Mosesso P., Barcellona P. S. (1990). Lonidamine: a non-mutagenic antitumor
agent. Carcinogenesis.

[44] Moraglio L., Brema F., Pastorino G., Martini M. C., Vallauri M. (1995). Combination
of Epirubicin and Lonidamine for Treatment of Advanced Breast Cancer. Tumori Journal..

[45] Aithal G. P., Ramsay L., Daly A. K., Sonchit N., Leathart J. B. S., Alexander G., Kenna G. J., Caldwell J., Day C. P. (2004). Hepatic
adducts, circulating antibodies, and cytokine polymorphisms in patients
with diclofenac hepatotoxicity. Hepatology.

[46] Ponsoda X., Bort R., Jover R., Gomez-Lechon M. J., Castell J. V. (1995). Molecular mechanism of diclofenac
hepatotoxicity: Association
of cell injury with oxidative metabolism and decrease in ATP levels. Toxicology in vitro.

[47] Helfgott S. M., Sandberg-Cook J., Zakim D., Nestler J. (1990). Diclofenac-associated
hepatotoxicity. Jama.

[48] Jafari S., Maleki-Dizaji N., Barar J., Barzegar-Jalali M., Rameshrad M., Adibkia K. (2016). Methylprednisolone acetate-loaded
hydroxyapatite nanoparticles as a potential drug delivery system for
treatment of rheumatoid arthritis: In vitro and in vivo evaluations. European Journal of Pharmaceutical Sciences.

[49] van
Everdingen A. A., Jacobs J. W., Siewertsz van Reesema D. R., Bijlsma J. W. (2002). Low-dose prednisone therapy for patients with early
active rheumatoid arthritis: clinical efficacy, disease-modifying
properties, and side effects: a randomized, double-blind, placebo-controlled
clinical trial. Annals of internal medicine.

[50] Murdoch W. R., Will G. (1962). Methylprednisolone acetate in intra-articular therapy. British Medical Journal.

[51] Bartok B., Firestein G. S. (2010). Fibroblast-like synoviocytes: key effector cells in
rheumatoid arthritis. Immunological reviews.

[52] Zhang L., Chang J., Zhao Y., Xu H., Wang T., Li Q., Xing L., Huang J., Wang Y., Liang Q. (2018). Fabrication
of a triptolide-loaded and poly-γ-glutamic acid-based amphiphilic
nanoparticle for the treatment of rheumatoid arthritis. Int. J. Nanomedicine..

[53] Fan D., Guo Q., Shen J., Zheng K., Lu C., Zhang G., Lu A., He X. (2018). The Effect of Triptolide in Rheumatoid Arthritis: From
Basic Research towards Clinical Translation. International Journal of Molecular Sciences.

[54] Li J., Shen F., Guan C., Wang W., Sun X., Fu X., Huang M., Jin J., Huang Z. (2014). Activation of Nrf2
protects against triptolide-induced hepatotoxicity. PLoS One..

[55] Mei Z., Li X., Wu Q., Hu S., Yang X. (2005). The research on the
anti-inflammatory activity and hepatotoxicity of triptolide-loaded
solid lipid nanoparticle. Pharmacol. Res..

