# Supplementary material for: Machine Learning Analysis of Cytotoxicity Determinants in Nanoparticle-Based Rheumatoid Arthritis Therapies
Source: Mol Pharm. 2025 Oct 23;22(11):6703–13. doi: 10.1021/acs.molpharmaceut.5c00661 (PMC12587385; doi:10.1021/acs.molpharmaceut.5c00661)
Supplement: Supplementary file 1 [file mp5c00661_si_001.pdf]

Supplementary Material for

Machine Learning Analysis of Cytotoxicity Determinants in Nanoparticle-Based Rheumatoid Arthritis Therapies

Elif Yildirim[1], Irem Cakir[1], Nazar Ileri-Ercan[1*]

[1]Chemical Engineering Department Middle East Technical University, 06800, Ankara, Turkiye

Table S1 shows the single-factor association rules with a minimum 70% confidence, and all rules stem from multiple sources. According to the results, Fe_3O_4, polydopamine, gold, and human serum albumin nanoparticles all exhibited non-toxic behavior. Magnetite (Fe_3O_4) [1-2], polydopamine [3-4], gold [5-6], and human serum albumin nanoparticles [7-8] have all been reported in the literature to show low toxicity and good biocompatibility. In addition, hyaluronic acid and cysteine materials resulted in non-toxic outcomes when applied as a coating/functional group. Notably, hyaluronic acid is also consistently reported in the literature as a biocompatible surface coating, validating the results [9-11]. Although our ARM analysis identified hyaluronic acid as potentially toxic when used as a core material, it was found to be non-toxic when applied as a surface coating, potentially due to differences in cellular interactions. Different from use as a core material, functionalization usually involves small amounts of hyaluronic acid at the material interface, avoiding toxic concentrations. Moreover, hyaluronic acid as a functionalized group is often chemically stabilized and less susceptible to rapid enzymatic degradation, avoiding low molecular weight byproducts. Positive zeta potentials above 20 mV are also reported to support cell viability. Moreover, healthy structural cells such as fibroblasts, chondrocytes, and endothelial cells exhibited high viability, consistent with expected low cytotoxicity in normal tissues. Certain synthesis methods, such as thin film hydration and sonication, along with Live/Dead and CCK-8 viability assays, were linked to higher cell viability across instances. This may be linked to milder nanoparticle formulations or assay sensitivity biases. Furthermore, nanoparticles without any encapsulated drugs were associated with non-toxicity. This is expected as drug incorporation often increases toxicity risks. Notably, dexamethasone (DEX) was associated with non-toxicity, consistent with literature information reporting low toxicity of dexamethasone at low doses [9], and reduced toxicity for short-term glucocorticoid treatments [10].

Table S1. Single-factor rules associated with non-cytotoxic outcomes (confidence ≥70%)

Support	Confidence	Lift	Count	Antecedent	Consequent
0.0136	1.0000	1.4	28	{Coat_FunctionalGroup=cysteine}	{Cell_Viability=non-cytotoxic}
0.0184	1.0000	1.4	38	{Synthesis_Method=Thin film hydration method}	{Cell_Viability=non-cytotoxic}
0.0214	1.0000	1.4	44	{Cell_Type=L929}	{Cell_Viability=non-cytotoxic}
0.0233	1.0000	1.4	48	{Cell_Type=Chondrocyte}	{Cell_Viability=non-cytotoxic}
0.0369	1.0000	1.4	76	{Core_Shell_Material=Fe3O4}	{Cell_Viability=non-cytotoxic}
0.0403	0.9881	1.4	83	{Core_Shell_Material=Polydopamine}	{Cell_Viability=non-cytotoxic}
0.0583	1.0000	1.4	120	{Type=H}	{Cell_Viability=non-cytotoxic}
0.0107	0.9565	1.3	22	{Assay_Indicator=Calcein AM/PI}	{Cell_Viability=non-cytotoxic}
0.0107	0.9565	1.3	22	{Viability_Assay=Live/Dead}	{Cell_Viability=non-cytotoxic}
0.0214	0.9362	1.3	44	{Cell_Type=HFLS-RA}	{Cell_Viability=non-cytotoxic}
0.0316	0.9286	1.3	65	{Cell_Tissue=Joint Cartilage}	{Cell_Viability=non-cytotoxic}
0.0383	0.9753	1.3	79	{Cell_Type=HUVEC}	{Cell_Viability=non-cytotoxic}
0.0413	0.9140	1.3	85	{NP_Concentration=no}	{Cell_Viability=non-cytotoxic}
0.0451	0.9490	1.3	93	{Cell_Morphology=Chondrocyte}	{Cell_Viability=non-cytotoxic}
0.0650	0.9241	1.3	134	{Core_Shell_Material=Au}	{Cell_Viability=non-cytotoxic}
0.0131	0.8438	1.2	27	{Viability_Assay=Trypan Blue Exclusion}	{Cell_Viability=non-cytotoxic}
0.0131	0.8438	1.2	27	{Assay_Indicator=Trypan Blue}	{Cell_Viability=non-cytotoxic}
0.0180	0.8605	1.2	37	{Cell_Tissue=liver}	{Cell_Viability=non-cytotoxic}
0.0214	0.8627	1.2	44	{Synthesis_Method=sonication}	{Cell_Viability=non-cytotoxic}
0.0238	0.8750	1.2	49	{Cell_Type=THP-1}	{Cell_Viability=non-cytotoxic}
0.0272	0.8889	1.2	56	{Coat_FunctionalGroup=HA}	{Cell_Viability=non-cytotoxic}
0.0447	0.8679	1.2	92	{Zeta_Potential=strongly positive}	{Cell_Viability=non-cytotoxic}
0.0471	0.8981	1.2	97	{Cell_Tissue=Bone Marrow}	{Cell_Viability=non-cytotoxic}
0.0471	0.8661	1.2	97	{Drug=DEX}	{Cell_Viability=non-cytotoxic}
0.0515	0.8480	1.2	106	{Core_Shell_Material=Human Serum Albumin}	{Cell_Viability=non-cytotoxic}
0.0631	0.8667	1.2	130	{Cell_Morphology=Endothelial}	{Cell_Viability=non-cytotoxic}
0.0631	0.8667	1.2	130	{Cell_Tissue=umbilical vein}	{Cell_Viability=non-cytotoxic}
0.3830	0.9069	1.2	789	{Drug=None}	{Cell_Viability=non-cytotoxic}
0.4126	0.9033	1.2	850	{Drug_Concentration=no}	{Cell_Viability=non-cytotoxic}

Table S2 shows double factor association rules with a minimum 70% confidence, and all rules stem from multiple sources. Biocompatible materials (Fe_3O_4, polydopamine, HSA, Au) previously identified in single-factor associations in favorable surface or size conditions are strongly linked to non-toxic outcomes. Firstly, non-cytotoxic outcomes were associated with Fe_3O_4 when combined with no drug encapsulation or weakly negative zeta potentials between -20 and 0 mV. Similarly, polydopamine nanoparticles with large TEM diameters above 150 nm and human serum albumin nanoparticles with TEM diameters between 50-150 nm exhibited high cell viability. In addition, gold nanoparticles with hydrodynamic diameters up to 50 nm were frequently linked with non-toxic outcomes.

Hyaluronic acid coatings paired with moderate exposure times between 24-48 hours were linked with low toxicity, along with PEG coatings combined with no drug encapsulation. Moreover, strongly positive zeta potentials above 20 mV combined with longer nanoparticle exposure times frequently resulted in high viability. Nanoparticles without drug encapsulation were consistently linked with non-cytotoxicity, which reflects the reduced potential of drug-induced stress. Low drug concentrations combined with fibroblast cells (L929) resulted in high viability, suggesting that minimizing the drug dosage facilitates the viability of sensitive structural cells commonly found in connective tissues. Cells

derived from joint cartilage and connective tissues consistently exhibited high cell viability, supporting the fact that nanoparticle formulations were tolerated well in these sensitive environments. Moreover, nanoparticles synthesized by thin film hydration and wet chemical routes were linked to high cell viability.

Table S2. Double-factor rules associated with non-cytotoxic outcomes (confidence ≥70%)

Support	Confidence	Lift	Count	Antecedent	Consequent
0.0136	1.0000	1.4	28	{Type=I, Coat_FunctionalGroup=cysteine}	{Cell_Viability=non-cytotoxic}
0.0107	1.0000	1.4	22	{Synthesis_Method=Thin film hydration method, NP_Concentration=high}	{Cell_Viability=non-cytotoxic}
0.0146	1.0000	1.4	30	{Drug_Concentration=low, Cell_Type=L929}	{Cell_Viability=non-cytotoxic}
0.0112	1.0000	1.4	23	{Drug_Concentration=no, Cell_Type=LPS activated THP-1}	{Cell_Viability=non-cytotoxic}
0.0131	1.0000	1.4	27	{Hydrodynamic_Diameter=0-50, Cell_Type=HFLS-RA}	{Cell_Viability=non-cytotoxic}
0.0233	1.0000	1.4	48	{Cell_Type=Chondrocyte, Viability_Assay=CCK-8}	{Cell_Viability=non-cytotoxic}
0.0117	1.0000	1.4	24	{TEM_Diameter=50-150, Cell_Type=THP-1}	{Cell_Viability=non-cytotoxic}
0.0238	1.0000	1.4	49	{Coat_FunctionalGroup=HA, Exposure_Time=24-48}	{Cell_Viability=non-cytotoxic}
0.0184	1.0000	1.4	38	{Coat_FunctionalGroup=PEG, Drug=None}	{Cell_Viability=non-cytotoxic}
0.0233	1.0000	1.4	48	{Cell_Tissue=Joint Cartilage, Viability_Assay=CCK-8}	{Cell_Viability=non-cytotoxic}
0.0238	1.0000	1.4	49	{Core_Shell_Material=Fe3O4, Zeta_Potential=weakly negative}	{Cell_Viability=non-cytotoxic}
0.0369	1.0000	1.4	76	{Core_Shell_Material=Fe3O4, Drug=None}	{Cell_Viability=non-cytotoxic}
0.0165	1.0000	1.4	34	{Drug=DEX, Cell_Type=HUVEC}	{Cell_Viability=non-cytotoxic}
0.0369	0.9870	1.4	76	{Core_Shell_Material=Polydopamine, TEM_Diameter=above 150}	{Cell_Viability=non-cytotoxic}
0.0204	1.0000	1.4	42	{Surface_Charge=Positive, Cell_Morphology=Chondrocyte}	{Cell_Viability=non-cytotoxic}
0.0131	1.0000	1.4	27	{TEM_Diameter=50-150, Cell_Morphology=Monocyte}	{Cell_Viability=non-cytotoxic}
0.0121	1.0000	1.4	25	{Zeta_Potential=strongly positive, Exposure_Time=above 48}	{Cell_Viability=non-cytotoxic}
0.0165	1.0000	1.4	34	{Drug=DEX, Cell_Morphology=Endothelial}	{Cell_Viability=non-cytotoxic}
0.0184	1.0000	1.4	38	{Drug=None, Synthesis_Method=wet chemical synthesis}	{Cell_Viability=non-cytotoxic}
0.0252	1.0000	1.4	52	{Type=H, Cell_Type=RAW 264.7}	{Cell_Viability=non-cytotoxic}
0.0248	1.0000	1.4	51	{Core_Shell_Material=Human Serum Albumin, TEM_Diameter=50-150}	{Cell_Viability=non-cytotoxic}
0.0146	1.0000	1.4	30	{Drug_Concentration=low, Cell_Tissue=Connective Tissue}	{Cell_Viability=non-cytotoxic}
0.0354	1.0000	1.4	73	{Core_Shell_Material=Au, Hydrodynamic_Diameter=0-50}	{Cell_Viability=non-cytotoxic}
0.1364	0.9305	1.3	281	{Drug=None, TEM_Diameter=50-150}	{Cell_Viability=non-cytotoxic}
0.1519	0.9343	1.3	313	{Drug=None, Hydrodynamic_Diameter=50-150}	{Cell_Viability=non-cytotoxic}
0.1636	0.9387	1.3	337	{Drug_Concentration=no, Hydrodynamic_Diameter=50-150}	{Cell_Viability=non-cytotoxic}
0.1612	0.9197	1.3	332	{Coat_FunctionalGroup=None, Drug=None}	{Cell_Viability=non-cytotoxic}
0.2204	0.9361	1.3	454	{Drug=None, NP_Concentration=medium}	{Cell_Viability=non-cytotoxic}
0.0117	0.9231	1.3	24	{Coat_FunctionalGroup=FA, Cell_Type=RAW 264.7}	{Cell_Viability=non-cytotoxic}

References

[1] Liu, G., Gao, J., Ai, H., & Chen, X. (2013). Applications and potential toxicity of magnetic iron oxide nanoparticles. *Small*, *9*(9-10), 1533-1545.

[2] Wu, L., & Shen, S. (2019). What potential do magnetic iron oxide nanoparticles have for the treatment of rheumatoid arthritis?. Nanomedicine, 14(8), 927-930.

[3] Chen, Y., Wang, Y., Jiang, X., Cai, J., Chen, Y., Huang, H., ... & Gao, M. (2022). Dimethylamino group modified polydopamine nanoparticles with positive charges to scavenge cell-free DNA for rheumatoid arthritis therapy. *Bioactive Materials*, *18*, 409-420.

[4] Fu, X., Song, Y., Feng, X., Liu, Z., Gao, W., Song, H., & Zhang, Q. (2024). Synergistic chemotherapy/PTT/oxygen enrichment by multifunctional liposomal polydopamine nanoparticles for rheumatoid arthritis treatment. *Asian Journal of Pharmaceutical Sciences*, *19*(1), 100885.

[5] Koushki, K., Keshavarz Shahbaz, S., Keshavarz, M., Bezsonov, E. E., Sathyapalan, T., & Sahebkar, A. (2021). Gold nanoparticles: multifaceted roles in the management of autoimmune disorders. *Biomolecules*, *11*(9), 1289.

[6] Fan, J., Li, X., Jin, M., & Li, C. (2024). Synthesis and biological evaluation of gold nanoparticles drug delivery system for anti-rheumatoid arthritis agents. *Journal of Drug Delivery Science and Technology*, *102*, 106402.

[7] Lyu, J., Wang, L., Bai, X., Du, X., Wei, J., Wang, J., ... & Zhong, Z. (2020). Treatment of rheumatoid arthritis by serum albumin nanoparticles coated with mannose to target neutrophils. *ACS Applied Materials & Interfaces*, *13*(1), 266-276.

[8] Yan, F., Li, H., Zhong, Z., Zhou, M., Lin, Y., Tang, C., & Li, C. (2019). Co-delivery of prednisolone and curcumin in human serum albumin nanoparticles for effective treatment of rheumatoid arthritis. *International Journal of Nanomedicine*, 9113-9125.

[9] Almalik, A., Alradwan, I., Majrashi, M. A., Alsaffar, B. A., Algarni, A. T., Alsuabeyl, M. S., ... & Alhasan, A. H. (2018). Cellular responses of hyaluronic acid-coated chitosan nanoparticles. *Toxicology Research*, *7*(5), 942-950.

[10] Wang, T., Hou, J., Su, C., Zhao, L., & Shi, Y. (2017). Hyaluronic acid-coated chitosan nanoparticles induce ROS-mediated tumor cell apoptosis and enhance antitumor efficiency by targeted drug delivery via CD44. *Journal of nanobiotechnology*, *15*, 1-12.

[11] Mirchandani, Y., Patravale, V. B., & Brijesh, S. (2022). Hyaluronic acid-coated solid lipid nanoparticles enhance antirheumatic activity and reduce toxicity of methotrexate. *Nanomedicine*, *17*(16), 1099-1114.

[12] Wang, Q., Jiang, J., Chen, W., Jiang, H., Zhang, Z., & Sun, X. (2016). Targeted delivery of low-dose dexamethasone using PCL–PEG micelles for effective treatment of rheumatoid arthritis. *Journal of Controlled Release*, *230*, 64-72.

[13] Laan, R. F., Jansen, T. L., & Van Riel, P. L. (1999). Glucocorticosteroids in the management of rheumatoid arthritis. *Rheumatology (Oxford, England)*, *38*(1), 6-12.
